# A path to Glucocorticoid Stewardship: a critical review of clinical recommendations for the treatment of systemic lupus erythematosus

**DOI:** 10.1093/rheumatology/keae041

**Published:** 2024-01-27

**Authors:** George Bertsias, Anca Askanase, Andrea Doria, Amit Saxena, Edward M Vital

**Affiliations:** Rheumatology and Clinical Immunology, University of Crete Medical School, Heraklion, Greece; Institute of Molecular Biology and Biotechnology, Foundation for Research and Technology—Hellas (FORTH), Heraklion, Greece; Division of Rheumatology, Department of Medicine, Columbia University Irving Medical Center, New York, NY, USA; Division of Rheumatology, Department of Medicine, University of Padova, Padova, Italy; Division of Rheumatology, Department of Medicine, NYU Langone Health, New York, NY, USA; Leeds Institute of Rheumatic and Musculoskeletal Medicine, University of Leeds, Leeds, UK; NIHR Leeds Biomedical Research Centre, Leeds Teaching Hospitals NHS Trust, Leeds, UK

**Keywords:** glucocorticoids, lupus erythematosus, systemic, lupus nephritis

## Abstract

Glucocorticoids (GCs) have revolutionized the management of SLE, providing patients with rapid symptomatic relief and preventing flares when maintained at low dosages. However, there are increasing concerns over GC-associated adverse effects and organ damage, which decrease patients’ quality of life (QOL) and increase healthcare costs. This highlights the need to balance effective GC use and minimize toxicity in patients with SLE. Herein, we provide an overview of the theoretical considerations and clinical evidence, in addition to the variations and similarities across nine national and eight international recommendations regarding the use of GCs across SLE manifestations and how these compare with real-world usage. In line with this, we propose possible actions toward the goal of GC Stewardship to improve the QOL for patients with lupus while managing the disease burden.

Rheumatology key messages

- Treatment recommendations in SLE recommend minimizing glucocorticoids (GC) use, owing to GC-associated adverse effects.
- There is a lack of sufficient guidance on effective GC-tapering regimens in clinical practice.
- These findings highlight the need for alternative treatment strategies to reduce GC dependency in SLE.

## Introduction

The discovery of glucocorticoids (GCs) resulted in the Nobel Prize in Physiology or Medicine in 1950, and they have since revolutionized the treatment of SLE [1], effectively reducing disease activity and preventing flares [1–4]. GCs can have effects on both genomic and non-genomic pathways, providing powerful anti-inflammatory and immunosuppressant actions [4]. GCs activate the genomic pathway at dosages as low as 2.5–5 mg/day prednisone equivalent and inhibit inflammatory cytokine gene transcription while increasing anti-inflammatory gene expression [1, 4]. Conversely, the non-genomic pathway is activated by higher dosages of prednisolone but more potently by methylprednisolone (MP) and dexamethasone [4]. This leads to reduced lymphocyte activity, providing rapid symptom relief [1, 4]. Despite its pivotal role in SLE management, GC use is overshadowed by concerns over adverse effects (AEs) and increased healthcare costs associated with GC-related toxicities [2, 5, 6]. Nevertheless, global studies suggest that up to 88% of patients with SLE are exposed to GCs [2, 3, 7–9], with many receiving them long term [2, 10, 11].

**Figure 1.**
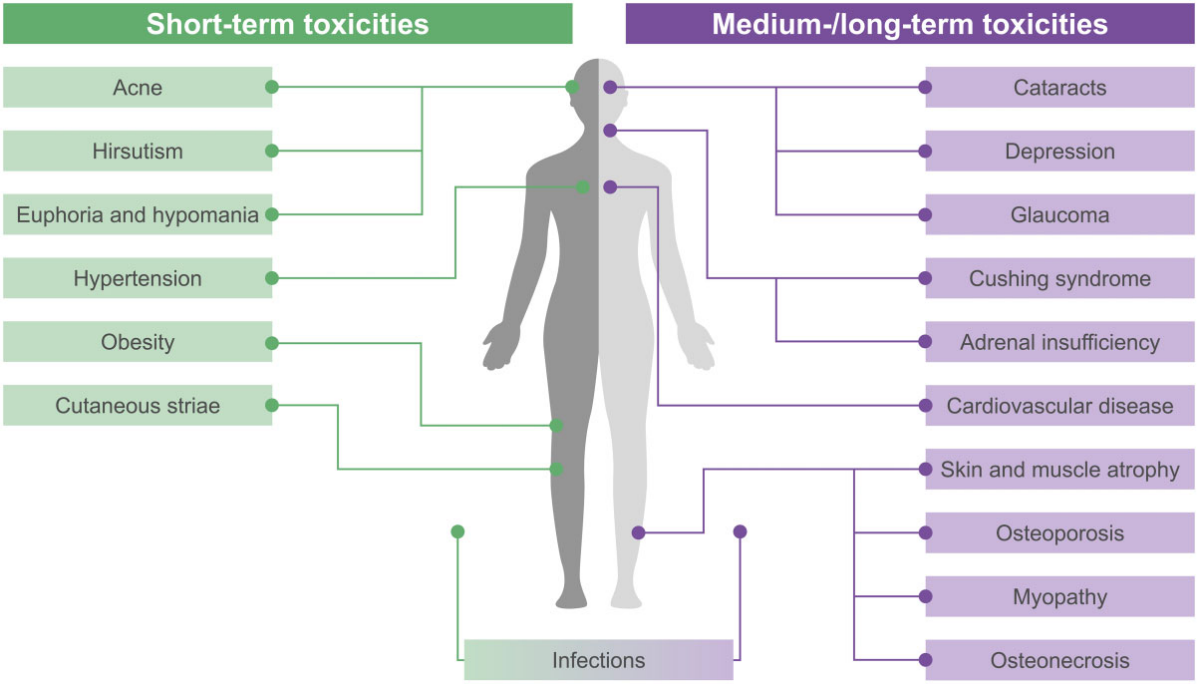
Short-, medium- and long-term toxicities associated with GC use in patients with SLE [5, 12–14]. GC: glucocorticoid

GC-associated AEs can occur across multiple organ systems, as shown in Fig. 1 [5, 12–14]. GC use contributes to organ damage accrual, quantified by the SLICC/ACR Damage Index (SDI), in patients with SLE, independent of disease activity [15, 16]. The association between GC dosages ≥7.5 mg/day and organ damage has been demonstrated in multiple studies and is acknowledged in treatment guidelines [8, 17, 18]. However, even dosages of ≥5–7.5 mg/day, as recommended for maintenance across treatment guidelines [18–21], are linked to AEs and long-term toxicity in patients with SLE [22]. Use of low-to-moderate GC dosages (≤30 mg/day prednisolone equivalent) is associated with osteoporosis [2, 17], diabetes [2], cataracts [2, 17], glaucoma [12], skin thinning [2], cardiovascular damage [17], weight gain [2] and infections [12], whereas higher dosages are additionally associated with myopathy [2], psychological and behavioural disturbances [2, 12], and osteonecrosis [2, 5]. Over long periods, cumulative GC exposure is associated with severe organ damage, including osteoporotic fractures, avascular necrosis, cataracts, coronary heart disease and diabetes [8, 23]. Adults with SLE have a 2- to 6-fold increase in relative risk of infection events compared with the general population, which is higher among those who have initiated GCs; this highlights the need to monitor patients with SLE for risk of GC-related infections [24]. GCs have also been shown to have AEs in children with SLE, including growth impairment [25, 26], and during pregnancy [25, 27–31]. Treatment guidelines note that GC use in the first trimester is associated with a moderately increased risk of cleft palate [25]. Furthermore, GC use is associated with potential risks of maternal complications, including hypertension, diabetes, preeclampsia, preterm birth and premature rupture of membranes, particularly at higher dosages of ≥10–20 mg/day [27–31].

The GC dosage during the first month of treatment is an independent predictor of GC burden during the following 11 months [32], suggesting the need to limit GC use early, which could be done by increasing immunosuppressant use from the beginning of treatment. A pilot study suggested that, after adjusting for disease activity, GC dosage >7.38 mg prednisone/day during the first year is a threshold associated with a 60% chance of permanent damage at 5 years in patients with SLE [33]. Higher cumulative GC exposure, as well as male sex and elevated SDI scores, has been shown to increase mortality risk in SLE, which may be explained by both GC toxicity and GC-associated comorbidities [34]. GCs at ≤7.5 mg/day may also negatively impact psychological health and quality of life (QOL) [35, 36].

GC use places a substantial burden on healthcare systems; patients with SLE receiving GCs tend to have higher healthcare resource utilization than non-GC users [6]. Although this could be confounded by the need for higher GC dosages in severe disease, aspects of this, such as osteoporosis, are directly attributable to GCs themselves and not lupus inflammation [6, 17]. In an insurance claims database study from the USA, patients receiving GCs ≤7.5 mg/day with concomitant immunosuppressants had lower incremental costs (the difference in total healthcare costs compared with patients not receiving GCs) than those receiving GCs ≤7.5 mg/day alone during a 1-year follow up ($1285 *vs* $2514, respectively) [6]. Therefore, strategies to reduce the use of GCs in SLE could improve patient care and healthcare resource utilization [6]. A key example is the concept of GC Stewardship, defined as a ‘systematic effort to prescribe and monitor glucocorticoids in a rational manner, while balancing benefit and potential risk, in patients who require this therapy’ [37]. Adoption of GC Stewardship strategies in patients with SLE will help to ensure rational and effective use of GCs without compromising patient outcomes [37].

This review aims to provide an overview of the variations and similarities across selected national and international recommendations regarding GC use across SLE manifestations, demonstrate how these compare with real-world usage and outline key areas for improvement in treatment guidelines to support GC Stewardship.

## Overview of the existing national and international guidance on GC use across SLE manifestations

Treatment recommendations published between 1997 and 2023 were identified through searches of PubMed and from the authors’ own suggestions. Only papers published in English, or those with English translations, were included. Nine national and eight international treatment guidelines were identified that discussed the use of GCs in SLE (*n *=* *8), LN (*n *=* *7) and cutaneous lupus erythematosus (CLE, *n *=* *2). Treatment recommendations were reviewed and compared on their guidance for GC use and are listed in Tables 1–3, respectively.

**Table 1.** Guidance on GC use in the treatment of SLE

	Initial therapy	Maintenance therapy	Treatment of severe manifestations	Guidance on tapering
APLAR 2021 [38]	Renal manifestations: MMF or i.v. pulse CYC combined with moderate-dosage GCs (∼0.6 mg/kg/day prednisolone)	–	Neuropsychiatric manifestations and other severe organ manifestations: moderate-to-high-dosage GCs (0.6–1 mg/kg/day prednisolone or equivalent) combined with CYC; additional pulses of i.v. MP may be needed for neuropsychiatric *vs* renal manifestations	Early combination with other IS or biological agents may allow use of lower GC dosages
Chinese Rheumatology Association, NCRC-DID and CSTAR 2020 [39]	Mild activity: GC use is generally not required; in patients refractory to HCQ or NSAIDs, use ≤10 mg/day prednisone or equivalent Moderate activity: 0.5–1 mg/kg/day prednisone or equivalent ± IS	–	≥1 mg/kg/day prednisone or equivalent combined with IS i.v. pulses of MP (500–1000 mg/day for ∼3 days) combined with IS in severe cases/lupus crises	Tapering or withdrawal of GCs may be considered for patients with long-term stable disease; avoid abrupt discontinuation Use of IS can reduce the cumulative dose of GCs and the risk of long-term AEs
EULAR 2023 [20]	Initial oral GC dose depends on disease activity i.v. pulses of MP may facilitate faster tapering of oral GCs	GCs should be minimized to ≤5 mg/day (prednisone equivalent) and withdrawn when possible Treatment should aim for remission or low disease activity and flare prevention using the lowest possible dosage	i.v. MP in cases of moderate-to-severe disease (125–1000 mg/day for 1–3 days) or proliferative LN (250–1000 mg/day for 1–3 days after excluding infections) Immunomodulating/IS agents may be included in the initial therapy in cases of organ-threatening disease	GCs should be used only as a bridging therapy, and the complete withdrawal of GCs is the optimal target Early initiation of IS and/or biologics can expedite tapering/discontinuation of GCs Gradual tapering of treatments should be considered in patients achieving sustained remission, starting with GC withdrawal
Mexican College of Rheumatology 2019 [19]	Initial dosage of GC (prednisone or equivalent) depends on disease activity Low activity: low dosages (<7.5 mg/day) Moderate activity: intermediate dosages (7.5–30 mg/day)	<7.5 mg/day	High dosages (30–100 mg/day or pulses >250 mg/day, usually i.v., for 1–5 days)	Begin tapering once reduction of disease activity or remission has been achieved (usually after 6 weeks of high dosages) Reduce by 10–20% every 7–15 days until 30 mg/day, then by 10% every 15 days until discontinued or continue with maintenance dosage
BSR 2018 [18]	Mild: oral prednisolone (≤20 mg/day) for ≤14 days to induce remission where local treatment is not sufficient/practical. For cutaneous manifestations, topical preparations should be used initially Moderate: ≤0.5 mg/kg/day prednisolone and/or intermittent i.m. MP (80–120 mg) or i.v. MP ≤250 mg	Mild: prednisolone ≤7.5 mg/day Moderate: IS often required to control active disease; reduce prednisolone dosages to the lowest possible maintenance dosage as disease activity improves	i.v. MP or high-dosage oral prednisolone (≤1 mg/kg/day) to induce remission on its own or as part of a treatment protocol with another IS	Prednisolone dosing should be reduced as disease activity improves and stopped, if possible, as other IS agents take effect over time
GLADEL-PANLAR 2018 [40]	Musculoskeletal manifestations: HCQ ± prednisone ≤7.5 mg Cutaneous manifestations: HCQ ± prednisone ≤7.5 mg Renal manifestations: HCQ ± prednisone ≤7.5 mg plus another IS agent recommended over GCs alone Cardiac manifestations: HCQ ± prednisone ≤7.5 mg plus colchicine	For LN, use MMF or AZA over CYC during maintenance therapy to minimize GC toxicity Prescribe GCs, if clinically needed, at the lowest possible dosage and for the shortest period of time, regardless of the manifestation	Severe pulmonary manifestations: i.v. GCs plus CYC and/or i.v. immunoglobulin and/or therapeutic plasma exchange and/or RTX over GCs alone Severe acute neuropsychiatric manifestations: high-dosage GCs plus CYC Severe haematological manifestations: high-dosage GCs ± IS or RTX	–
CPG-SLE (Spain) 2016 [21]	Prednisone dosage ≤30 mg/day in LN or other manifestations, but the dosage should be individualized	If maintenance is required, prednisone ≤5 mg/day	Severe outbreaks: adjuvant therapy with pulses of MP is recommended; use of <1000 mg pulses is suggested, but no specific dosage is recommended	Quick reduction of prednisone to 5 mg/day is recommended within 6 months, with full withdrawal as soon as possible
			Neuropsychiatric SLE: i.v. CYC combined with prednisone or MP is recommended over GCs alone	
ACR Ad Hoc Committee on SLE 1999 [41]	–	Mild: usually does not require systemic GC treatment If required to improve QOL, low-dosage daily or alternate-day GC (≤10 mg/day prednisone or equivalent) can be used, but monitor for GC toxicity Refer to a rheumatologist if GCs are required	High-dosage GCs (40–60 mg/day prednisone or i.v. pulses of MP) ≤1 g/day for 3 days) for severe organ-threatening disease or ≤20 mg/day prednisone or equivalent for refractory serositis Dosage and mode of administration depend on nature and severity of disease	–

AE: adverse effect; APLAR: Asia Pacific League of Associations for Rheumatology; BSR: British Society for Rheumatology; CPG: Clinical Practice Guideline; CSTAR: Chinese Systemic Lupus Erythematosus Treatment and Research Group; GC: glucocorticoid; GLADEL-PANLAR: Grupo Latino Americano de Estudio del Lupus–Pan-American League of Associations of Rheumatology; IS: immunosuppressant; MP: methylprednisolone; NCRC-DID: National Clinical Research Center for Dermatologic and Immunologic Diseases; QOL: quality of life; RTX: rituximab.

**Table 2.** Guidance on GC use in the treatment of LN

	Initial therapy	Maintenance therapy	Treatment of severe manifestations	Guidance on tapering
KDIGO clinical practice guidelines for the management of glomerular diseases 2021 [25]	Class III or IV: initial GC dosage of 0.5–0.6 mg/kg/day (max 40 mg) following a short course of MP pulses may be considered during the initial treatment of active LN when both the kidney and extrarenal disease manifestations show satisfactory improvement plus either low-dosage i.v. CYC or MPAA	≤7.5 mg/day, and preferably as low as possible Class III or IV: prednisone <5–7.5 mg/day combined with MPAA	Active Class III or IV, with or without a membranous component: GCs plus either low-dosage i.v. CYC or MPAA Class V with nephrotic syndrome: combined IS treatment with GC and one other agent	Class III or IV: GCs should be tapered to the lowest possible dosage during maintenance (except when required for extrarenal manifestations) and may be discontinued after maintenance of complete clinical renal response for ≥12 months
EULAR/ERA-EDTA 2020 [42]	Class III or IV: MMF (or MPA at equivalent dosage) or CYC in conjunction with i.v. pulses of MP (total dose 500–2500 mg, depending on disease severity) is recommended to reduce cumulative GC dose, followed by oral prednisone (0.3–0.5 mg/kg/day) for up to 4 weeks	Active proliferative LN: no or low-dosage GCs (<7.5 mg/day) combined with MMF or AZA	Class III or IV (±V): GCs combined with MMF (or MPA at equivalent dosage) or low-dosage i.v. CYC Class V: pulse i.v. MP (total dose 500–2500 mg, depending on disease severity) followed by oral prednisone (20 mg/day) combined with MMF (or MPA)	After i.v. MP pulses and oral prednisone for up to 4 weeks, taper prednisone to ≤7.5 mg/day by 3–6 months Class V: oral prednisone may be tapered to ≤5 mg/day by 3 months Belimumab may be used to facilitate GC sparing
Brazilian Society of Rheumatology 2015 [28]	Class III or IV: CYC or MMF in conjunction with i.v. MP pulse (0.5–1.0 g/day for 3 days) followed by 0.5–1.0 mg/kg/day prednisone for 3–4 weeks, with subsequent reduction to 5–10 mg/day after 6 months	Class III–V: low-dosage GCs (<10 mg/day) in addition to AZA or MMF in combination with HCQ and adjuvant therapy are indicated for patients who have achieved complete or partial remission in the induction phase	Prescribe higher dosages of prednisone (1.0 mg/kg/day) to patients with worse prognosis factors	Class III–V: reduce GC dosage progressively and discontinue, if possible, ideally after achieving a complete and sustained remission; owing to the high frequency of AEs, every effort should be made to reduce the daily GC dosage
Asian Lupus Nephritis Network 2014 [43]	Mild/moderate disease: initial treatment with moderate-dosage GCs alone or in combination with AZA or MMF	–	Initial combined therapy with prednisolone 0.8 mg/kg/day and MMF or CYC Pulse MP (0.5–1.0 g/day for 3 days) when renal biopsy shows crescentic involvement >10%, or there is renal function deterioration, followed by oral prednisolone at 0.5–0.6 mg/kg daily	Severe disease: taper GCs after 2 weeks except in patients with no sign of improvement, aiming to reach <20 mg/day after 3 months and ≤7.5 mg/day after 6 months
ACR 2012 [29]	Class III or IV: pulse i.v. MP (500–1000 mg/day for 3 days) combined with IS, followed by daily oral GCs (0.5–1 mg/kg/day)	Class III or IV: MMF or AZA ± low-dosage GCs	Class IV or V with cellular crescents: combine CYC or MMF with pulse i.v. MP (500–1000 mg/day for 3 days), followed by daily oral GCs (1 mg/kg/day) Class V: MMF and 0.5 mg/kg/day prednisone for 6 months. If not improved, monthly CYC for 6 months, plus GC pulses, followed by prednisone 0.5–1.0 mg/kg/day	Class III or IV: GCs should be tapered to the minimal amount required to control disease; however, there are insufficient data to recommend a specific steroid taper as nephritis and extrarenal manifestations vary across patients
Dutch Working Party on SLE (proliferative LN guidelines) 2012 [44]	MMF combined with 1 mg/kg/day prednisone (maximum 60 mg/day) or CYC with i.v. MP (750 mg/day for 3 days) followed by prednisone 0.5–1.0 mg/kg/day	–	–	If MMF plus prednisone is used as induction therapy, taper dosages by 10 mg every 4 weeks until 20 mg/day, then by 5 mg every 4 weeks to 10 mg/day If CYC plus MP pulses is used as induction therapy, taper prednisone dosages after 4 weeks; taper dosages every 2 weeks by 2.5 mg to 5–7.5 mg at 30 months 4 years after induction therapy, prednisone can be tapered to 10 mg every other day
SEMI-SEN guidelines 2012 [30]	Class II:^a^ GC dosages (≤0.5 mg/kg/day) with/without IS agents (e.g. AZA, MMF), as GC-sparing drugs for 6–12 months	After achieving at least partial response during induction therapy: Class III/IV: low-dosage GCs (≤10 mg/day) and MMF should be used Class V: low-dosage GCs (≤10 mg/day) and either MMF, CNIs or AZA	Class III/IV: ≤1 mg/kg/day prednisone (maximum 60 mg/day), combined with either CYC or MMF,^b^ although smaller GC dosages (≤0.5 mg/kg/day) can be used with i.v. MP pulses (250–1000 mg) Class V: ≤1 mg/kg/day prednisone (maximum 60 mg/day), combined with either CYC or MMF,^b^ CNIs or AZA	Prednisone dosages should be rapidly reduced until reaching a maintenance dosage ≤5 mg/day, or even halting this treatment based on disease activity

a In the presence of significant proteinuria despite renal protective treatment and/or renal function deterioration not attributable to functional factors.

b Or enteric-coated mycophenolate sodium. AE: adverse effect; CNI: calcineurin inhibitor; ERA-EDTA: European Renal Association–European Dialysis and Transplant Association; GC: glucocorticoid; IS: immunosuppressant; KDIGO: Kidney Disease: Improving Global Outcomes; MP: methylprednisolone; MPA: mycophenolic acid; MPAA: mycophenolic acid analogs; SEMI-SEN: Spanish Society of Internal Medicine–Spanish Society of Nephrology.

**Table 3.** Guidance on GC use in the treatment of CLE

	Initial therapy	Maintenance therapy	Treatment of severe manifestations	Guidance on tapering
British Association of Dermatologists 2021 [27]	Consider potent topical GCs as first-line monotherapy in patients with localized CLE (for ≤4 weeks) or as an adjuvant to systemic therapy if widespread cutaneous and/or SLE involvement Short-term and tapering courses of systemic GCs considered for flares	Consider a twice-weekly dosage of potent topical GCs for maintenance in patients who respond to topical GCs or CNIs and review effectiveness at 3–6 months	Concomitant, short-term and tapering courses of systemic GCs for severe/disseminated disease or subtypes with greatest risk of scarring	–
S2K guidelines 2021 [45]	Topical GCs are recommended for treating circumscribed CLE lesions, but duration of use should be limited to the shortest possible time because of the AE profile of topical GCs, in addition to the location of the skin lesions For severe or disseminated CLE lesions, systemic GCs are recommended as first-line treatment in addition to AMs for a limited period of time	–	For patients with extensive lesions, an inclination to scarring or insufficient response, a combination of topical GCs with a systemic treatment (e.g. AMs) is recommended	Systemic GCs should only be prescribed for limited periods of time, and the dosage kept as low as possible Systemic GCs should be tapered off as soon as possible, and complete discontinuation is always the goal

AE: adverse effect; AM: antimalarial; CLE: cutaneous lupus erythematosus; CNI: calcineurin inhibitor; GC: glucocorticoid.

### Initial treatment

Overall, recommendations agreed on suggesting an initial short course of GCs for immediate therapeutic effect, if needed, followed by a lower maintenance dosage [18, 25, 28, 29, 42]. A range of initial dosages of GCs were suggested across treatment recommendations and SLE manifestations (Tables 1–3), with many recommending that initial dosage should be tailored to an individual’s disease severity.

In patients with SLE, short courses of i.v. MP pulses (dosages range from 125 to 1000 mg/day for ∼3 days) are typically reserved for the treatment of moderate or severe SLE [18–21, 39]. Initial oral GC dosages stated across SLE treatment guidelines typically range from ≤7.5 mg/day to 1 mg/kg/day [18, 19, 21, 38–41]. Chinese guidelines suggest considering dosages ≥1 mg/kg/day prednisone or equivalent in combination with immunosuppressants in patients with severe active SLE [39]. Recommendations from Latin America suggest that initial treatment with low-dosage GCs (≤7.5 mg/day) is sufficient in adults with SLE with low disease activity [19, 40].

In patients with Class III or IV LN, i.v. MP pulses (dosages ranging from 250 to 1000 mg/day for ∼3 days) are generally recommended as initial treatment to induce remission and are typically followed by a course of moderate- or high-dosage oral GCs (0.3–1.0 mg/kg/day for up to 4 weeks) then gradually tapered [25, 28–30, 42–44]. Guidance from the joint EULAR and European Renal Association–European Dialysis and Transplant Association (ERA-EDTA) recommends a total dose of i.v. MP of 500–2500 mg, depending on disease severity [42]. Treatment recommendations from Spain and the Netherlands propose a maximum initial prednisone dosage of 60 mg/day in patients with LN [30, 44].

### Maintenance therapy

Use of the lowest possible dosage is widely recommended as maintenance therapy across SLE manifestations (Tables 1–3). There is consensus that GCs should only be prescribed for limited periods of time and be tapered as soon as possible to the lowest dosage required to control disease activity or completely discontinued [18–21, 25, 28–30, 39, 40, 42, 45]. There is also consensus from most guidelines that no more than >7.5 mg/day, and no more than >5 mg/day in the updated EULAR 2023 recommendations, is advised for GC maintenance therapy [18–20, 25, 42]. However, specific dosage recommendations vary across guidelines.

In patients with SLE, maintenance treatment recommendations range from ≤5 mg/day, if required, in guidance from EULAR [20] and Spain [21], to ≤10 mg/day in patients with mild, non-refractory SLE by the ACR Ad Hoc Committee on SLE [41]. Guidance from China and ACR state that systemic GCs are not typically needed for patients with mild SLE [39, 41] but can be used in patients refractory to HCQ or NSAIDs [39] or, in some cases, to improve QOL [41]. The latest EULAR recommendations suggest GCs should only be used as ‘bridging therapy’ in SLE; the lowest possible dose of GCs should be prescribed for the shortest possible period and at a maximum maintenance dosage of 5 mg/day in view of the detrimental impact of long-term use and the approval of new agents with GC-sparing effects [20].

Regarding LN, Brazilian and Spanish recommendations suggest a maintenance dosage of ≤10 mg/day in patients with Class III–V LN [28, 30], whereas the Kidney Disease: Improving Global Outcomes (KDIGO) guidelines and EULAR/ERA-EDTA recommendations suggest ≤7.5 mg/day as maintenance in patients with Class III–V LN [25, 42].

### Guidance on monitoring for GC-related AEs

Given the significant toxicity associated with GC use, many treatment recommendations advise monitoring treatment-associated AEs [19, 25, 27, 38, 41]. Generally, guidance suggests monitoring patients receiving GCs for cardiovascular and bone health, weight, metabolic parameters, and ophthalmological assessments [19, 21, 25, 38, 41, 46].

In patients with SLE, EULAR and the Asia Pacific League of Associations for Rheumatology (APLAR) recommend the use of the lowest possible GC dosage to minimize potential harm, including cardiovascular events [47] and infection risk [38]. Although the association between GC use and infection is discussed across several recommendations [18, 20, 21, 38, 39], only guidelines from Mexico and ACR advise monitoring for GC-related infections and recommend vaccination in patients receiving GCs [19, 41].

Guidance from ACR and APLAR outline the importance of regular assessment of cardiovascular and osteoporotic risk factors in patients with SLE receiving GCs [38, 41]. ACR guidelines for GC-induced osteoporosis recommend that all adults initiating or continuing GC therapy ≥2.5 mg/day for >3 months should undergo initial clinical fracture risk assessment and bone mineral density testing, with reassessments every 1–2 years [46]. Moreover, they recommend use of either bisphosphonates, denosumab or parathyroid hormone analogues in adults receiving high-dosage GCs (initial dosage ≥30 mg/day for >30 days or cumulative dose ≥5 g in 1 year) [46]. Mexican guidelines recommend calcium and vitamin D supplementation in patients receiving high dosages of prednisone for >3 months [19].

In Dutch and Spanish LN guidelines, calcium and vitamin D supplementation is also recommended for patients receiving GCs in addition to bisphosphonates, especially in patients aged >50 years, or in patients with a history of fractures [30, 44].

For patients with CLE, the British recommendations advise considering comorbidities in patients and the benefit:risk ratio of systemic GCs before initiating treatment [27]. Additionally, they state that patients receiving long-term (>3 weeks) or frequent courses (3–4 per year) of GCs should be regularly monitored to prevent GC-induced osteoporosis or adrenal insufficiency [27]. German guidelines advise daily vitamin D supplementation, particularly in patients receiving systemic GCs [45].

### Alternative therapeutic approaches to minimize GC use

Most treatment recommendations recognize the need for alternative strategies to minimize GC use and advise using steroid-sparing strategies to facilitate this across SLE manifestations [18, 20, 25, 28, 30, 38, 39, 41–43]. They suggest that early use of immunosuppressants or biologic agents may facilitate lower dosages of GCs, expedite tapering and reduce the risk of long-term AEs [20, 38, 39]. Provided there are no contraindications, HCQ is recommended to prevent flares in all patients with lupus [18, 20, 25, 28, 30, 38, 39, 41, 42] and has demonstrated a GC-sparing effect [18]. While acknowledging evidence of a GC-sparing effect with belimumab, APLAR did not recommend use of belimumab as a first-line agent in patients with SLE owing to cost-effectiveness concerns [38]. Since the publication of the majority of these recommendations, anifrolumab has been approved for the management of moderate-to-severe SLE [48] and, along with belimumab, is recommended by EULAR to control disease activity and facilitate GC tapering, with no hierarchy between both treatments [20].

### Guidance on GC tapering

Although many treatment recommendations advise on the need for GC tapering, specific guidance on effective regimens is lacking (Tables 1–3). For patients with SLE, Spanish guidelines recommend reduction of GCs to 5 mg/day within 6 months and full withdrawal as soon as possible [21]. Mexican guidelines suggest initiating GC tapering once disease activity is reduced or remission achieved, typically starting 6 weeks after GC use until discontinuation, or a maintenance dosage <7.5 mg/day is reached [19].

For LN, EULAR/ERA-EDTA guidelines recommend that patients receive an initial oral prednisone dosage of 0.3–0.5 mg/kg/day, reduced to ≤7.5 mg/day by 3–6 months [42]. The KDIGO guidelines provide comprehensive guidance on GC tapering, in which examples of standard-/moderate-/reduced-dosage GC regimens for patients with LN are provided [25].

The concept of alternate-day GC treatment regimens is explored in two treatment recommendations. ACR guidance for mild SLE recommends daily or alternate-day GCs (≤10 mg/day prednisone or equivalent), if needed, to improve QOL [41]. For LN, the Dutch guidelines recommend tapering prednisone dosages 4 years post-initiation of induction therapy to 10 mg every other day [44]. However, it should be noted that alternate-day oral GC regimens have been associated with relapses [18].

### GC use in special populations: patients with childhood-onset SLE, LN and CLE

In addition to the adult treatment recommendations reviewed in Tables 1–3, guidance was also reviewed from the Single Hub and Access Point for Paediatric Rheumatology in Europe (SHARE) initiative [26]. Short-term GC use in childhood-onset SLE, LN and CLE is supported by current treatment recommendations [25–28, 40, 42]. Recommendations across manifestations suggest adding disease-modifying drugs to permit GC tapering in children with lupus [26, 27]. Moreover, recommendations from the SHARE initiative state that prepubertal and peripubertal patients with SLE receiving a high cumulative dose of GCs must be proactively assessed for growth impairment [26].

Guidance from KDIGO states that when devising a therapy plan for children with LN, considerations must be made regarding dosage adjustment, growth, fertility and psychosocial factors [25]. Specific guidance for appropriate GC dosages in childhood-onset LN is provided in Latin American guidelines, which advise against prolonged GC exposure [28, 40] and recommend that induction with high-dosage GCs (prednisone 1–2 mg/kg/day, maximum 60 mg/day) combined with another immunosuppressive agent is preferable to using high-dosage GCs alone; GCs are not recommended for maintenance therapy [40]. Brazilian guidelines recommend i.v. MP 10–30 mg/kg/day for 3 days followed by prednisone 0.5–1.0 mg/kg/day for 3–4 weeks, with progressive reduction, aiming to achieve dosages of 5–10 mg/day after 6 months [28]. With accumulating data on the efficacy and GC-sparing role of immunosuppressive medications, there is a shift toward reducing GC exposure in paediatric patients with lupus [25].

### GC use in special populations: pregnancy in adult SLE, LN and CLE

In addition to the aforementioned guidelines, several pregnancy-specific treatment guidelines were reviewed for their guidance on GC use across SLE manifestations [31, 49–51]. Guidelines advise that pregnancy should be avoided during active SLE and LN until disease activity remains stable for ≥6 months without vital organ damage [28, 39]. There is a consensus supporting GC use to prevent or manage flares or active disease during pregnancy in patients with SLE, LN and CLE [25, 28–31, 39, 45, 49]. Prednisone and MP are recommended when necessary for use in pregnancy as they are largely inactivated by the placenta [30, 49, 50]. However, fluorinated GCs, such as betamethasone and dexamethasone [30], should be avoided during the first trimester because of their associated risks, such as impaired fetus growth, and should only be used when the benefit outweighs the risk for the mother and child [49, 52, 53]. Guidelines agree that the lowest GC dosage required to suppress disease activity should be used during pregnancy [27, 29, 45]. However, the use of high-dosage GCs as i.v. pulse therapy during pregnancy is recognized by EULAR as an effective intervention to manage moderate-to-severe flares [31] and in Spanish LN guidelines in severe situations [30]. Treatment options during pregnancy are gradually expanding, allowing greater opportunities for use of GC-sparing strategies in pregnant women with SLE [51].

CLE recommendations provide stricter and more specific guidance on GC dosages during pregnancy than SLE and LN recommendations [27, 29, 31, 45]. German CLE recommendations advise against using regular dosages >7.5 mg/day during pregnancy, if possible [45], and the British Association of Dermatologists recommends <10 mg/day where systemic GCs are needed for severe or active CLE during pregnancy [27].

## Real-world use of GCs

GCs are the mainstay of SLE management, with little change in their use over the past few decades [7]. Real-world data from Japan highlight that GCs and NSAIDs are the most frequently prescribed therapies for patients with newly diagnosed SLE [11]. Despite recommendations advising the use of the lowest possible dosage of GCs, real-world evidence of treatment patterns suggests that long-term use of GC dosages ≥7.5 mg/day is common in clinical practice [11, 17, 54]. In an insurance claims database analysis from the USA of 27 033 patients diagnosed with SLE between January 2012 and May 2018 who had two or more pharmacy prescriptions for GCs, 23.3% were prescribed an average dosage of 7.5–<15 mg/day, and 59.6% were prescribed an average dosage of ≥15 mg/day [54]. Moreover, data from the multicentre Asia Pacific Lupus Collaboration cohort demonstrated that >50% of patients remained on GC treatment following >4 years of treatment, highlighting the chronic use of GCs in clinical practice [10]. Studies have identified significant variation in GC use between treatment centres, even within countries or regions [7, 55]. Variations have also been observed in clinical trials; outputs from the anifrolumab TULIP-1 trial suggest that there are variations in GC treatment and prescribing behaviour across Eastern and Western Europe [56].

### Predictors of GC use

Significant variation in GC use has been observed across different patient ancestries; in a single-centre prospective study from Cleveland, USA (*N* = 173), patients with non-European ancestry were more likely to receive GCs (73%) than patients with European ancestry (34%), and more likely to utilize chronic GCs after adjusting for disease activity and other medications [57]. Data from the SLICC inception cohort revealed additional baseline clinical and demographic factors in patients who experienced greater GC exposure, such as younger age, shorter disease duration and male sex [7]. Furthermore, patients with Hispanic ethnicity, or Asian or African ancestry were more likely to receive GCs than patients with European ancestry, and patients with Hispanic ethnicity were more likely to receive higher GC dosages [7]. In a multivariable analysis, there was a notable effect of the treatment centre where patients received their care, indicating inconsistency in prescribing practice seemingly regardless of the other characteristics assessed [7]. Such data are important to physicians for identifying patients at higher risk of toxicity and those who would benefit from GC-sparing approaches.

## Discussion

Treatment guidelines and recommendations in SLE recognize the risks associated with GCs and highlight the need to use the lowest possible dosage and withdraw GC treatment altogether, if possible; however, there is a lack of sufficient guidance on how to effectively taper GCs in clinical practice. Despite most guidelines recommending dosages of ≤5–7.5 mg/day, even dosages as low as <5 mg/day have been shown to contribute to long-term damage accrual [22]. Given the need for alternative strategies to reduce GC dependency to successfully implement GC tapering in patients with SLE, consensus is needed across treatment recommendations on the best management approaches.

GC Stewardship aims to minimize GC overuse and spare patients from irreversible AEs [37]. GC Stewardship methods include pre-prescription screening, rational prescription, medical care during GC use, tapering of GCs and appropriate monitoring following GC discontinuation [37]. Tapering is feasible in patients with SLE where remission or low disease activity is present [58, 59]. In these patients, GC withdrawal has been associated with reduced damage accrual and no significant increases in the risk of flares compared with patients receiving GCs as part of a maintenance regimen [16, 58, 59]. Additionally, gradual tapering can lead to discontinuation of GCs, even in patients with prior severe organ involvement [60, 61]. GC use has been reduced in other therapy areas, such as RA and IBD, where the development of advanced therapies allied with guideline reform has brought about a paradigm shift in GC prescribing [62–65].

There is increasing evidence that immunomodulatory therapy may improve clinical outcomes and reduce reliance on GCs [3, 66]. Combination therapy with immunosuppressants, antimalarials or biologic treatments can enable GC dosage tapering or eventual withdrawal [4, 66–69]. While the GC-sparing effect of HCQ is acknowledged in British treatment recommendations [18], the observed effect has been modest in clinical practice. Results from several clinical trials have demonstrated that combining immunosuppressants, such as mycophenolate sodium or MMF and voclosporin, with GCs may facilitate lower GC dosages without compromising clinical outcomes in patients with LN [70, 71]. Moreover, a single-centre cohort study demonstrated achievement of remission in patients with LN with combined rituximab and MMF without oral GCs [72]. These findings suggest that introducing advanced therapies may reduce the GC dosage required for effective treatment and even lead to GC-free regimens without compromising clinical outcomes in patients with lupus.

### Barriers to GC tapering

A key barrier to implementing GC tapering in clinical practice is the marked heterogeneity in access to affordable medical services and advanced therapies across the globe [43, 73, 74]. In specialist care across Asia, socioeconomic factors and reimbursement systems vary greatly and have a significant impact on the management of SLE manifestations [43]. In this region, financial limitations, education level and adherence to prescribed regimens, organization of healthcare structure and delivery, and infection risks imposed by environment and climate can all be strong determinants of the access to evidence-based standard of care and treatment decisions [43]. While several treatment recommendations acknowledge the GC-sparing effect of immunosuppressants and advanced therapies [20, 38, 39], socioeconomic barriers may prevent accessibility to these therapies, ultimately resulting in chronic GC use [43]. For example, oral GCs may be a favoured treatment option for uninsured individuals or in poorer countries due to their relatively low cost [7], while the cost of GC-sparing agents, such as biologics, is a key barrier preventing their recommendation more widely in the Asia-Pacific region [38] and in low- and middle-income countries [75]. This highlights the need for changes to healthcare policies to provide reimbursement of GC-sparing agents to help make a meaningful impact on the reduction of GC dependency in clinical practice. Furthermore, to address the observed variability in GC prescribing and use, the SLICC consortium suggests that international consensus guidelines for GC use in different clinical situations are needed [7].

Lack of representation across clinical trials and treatment recommendations is another key barrier for GC Stewardship in certain populations [38, 76]. For example, there is a lack of country-specific clinical trial data from Brazil on treatment options for SLE and LN [76]. Moreover, there are insufficient data regarding efficacy of biologics as first-line therapies for LN in Asian populations, thus limiting their use and further contributing to GC dependency [38]. This highlights the need for medical societies and health institutions to establish country-specific SLE/LN data registries to characterize the disease course and develop strategies for affected populations [76]. Additionally, evidence indicates demographic variables, such as sex, age and ancestry, are strongly associated with GC exposure [7]. While these variables may be a source of health inequality, they could be considered as part of GC Stewardship strategies.

Given the rapidly changing treatment landscape in SLE, several treatment recommendations are now outdated and do not include the latest therapies [41, 76]. Moreover, many recommendations are heavily based on expert opinion [18, 20, 25, 28, 29, 38, 43, 44]; this highlights the need for regular evidence-based updates to treatment recommendations to ensure optimum patient care. In addition, there is an unmet need for provider education on the evolving treatment paradigm in lupus care; this would ensure that providers are aware of the latest advanced therapies and GC-sparing agents and therefore may contribute to GC Stewardship implementation.

While there is consensus on the need to minimize GC use where possible, specific guidance on tapering regimens is required, as rapidly decreasing GC dosages may precipitate a flare or adrenal insufficiency [4]. A recent study found a significantly lower risk of flare when GCs were tapered over a 12- to 24-month period compared with rapid (<3 months) tapering in patients with SLE [61]. Further guidance is needed in treatment recommendations to standardize GC dosage protocols and enable patients with SLE to be safely tapered off GCs without compromising clinical outcomes. To achieve this, a dedicated taskforce has been assembled to generate consensus-based GC tapering recommendations and example tapering regimens, using a modified Delphi methodology. Further potential strategies to facilitate successful GC Stewardship in clinical practice are outlined in Table 4.

**Table 4.** Strategies that support GC Stewardship

Strategy	Explanation	Examples	Reference(s)
Pre-prescription screening and rational prescription	It is important for physicians to have a clear understanding of the aim of GC prescription Physicians should consider the benefit:risk ratio, possible limitations and associated costs before initiating GCs	Ensuring clarity of indication for use Providing patient counselling and education Performing medical and endocrine screening	Kalra *et al.* [37]
Medical care during GC use	Owing to known AEs associated with GC use, patients should be monitored for GC-related AEs Appropriate management of GC-induced complications must be introduced in a timely manner	Ensuring that prescribing instructions are clearly mentioned to patients Performing regular monitoring (e.g. metabolic and endocrine assessments) for potential AEs related to GC use	Kalra *et al.* [37]
Use of immunomodulatory therapy to reduce GC dependence	Combination therapy with other agents can facilitate lower doses of GCs	AMs (e.g. HCQ) Immunosuppressants (e.g. MMF, AZA, MTX) Biologics (e.g. belimumab, anifrolumab, rituximab)	Apostolopoulos and Morand [3] Rua-Figueroa Fernández de Larrinoa *et al.* [66] Porta *et al.* [4] Gatto *et al.* [67]
Tapering, when in remission, and complete withdrawal	Studies have shown that GC tapering and complete withdrawal is an achievable goal for patients with SLE Duration of therapy must be considered when planning a tapering strategy	GC tapering is feasible in patients with low disease activity (or in remission) If duration of GC therapy is >3 weeks, tapering must be gradual to allow recovery of the HPA axis and avoid adrenal failure Complete withdrawal can be attempted after long-term remission or LLDAS	Tselios *et al.* [58] Tani *et al.* [59] Zen *et al.* [16] Nakai *et al.* [60] Kalra *et al.* [37]
Monitoring following discontinuation	Responsibility of GC Stewardship should continue even after cessation of treatment; therefore, patients should continue to be monitored to avoid long-term AEs	Performing medical and endocrine screening Close disease monitoring for flares Mitigation of long-term AEs	Kalra *et al.* [37] Tani *et al.* [59]

AE: adverse effect; AM: antimalarial; GC: glucocorticoid; HPA: hypothalamic-pituitary-adrenal; LLDAS: Lupus Low Disease Activity State.

Clinical research may be required to elucidate the factors that facilitate GC tapering; studies on the association between disease activity and tapering ability may help to identify candidates eligible for GC withdrawal with minimal safety impacts. Additionally, training to normalize GC-tapering practices may result in a more standardized approach [56].

## Conclusion

There is a clear need to educate patients and physicians on the risks associated with cumulative GC exposure and the measures that can be taken to reduce their impact. The concept of GC Stewardship should be encouraged in clinical practice; GCs should only be used when clinically appropriate and at the lowest dosage possible, with a consensus on appropriate strategies to effectively taper GC dosages urgently needed. Key next steps to ensure GC Stewardship include the generation of evidence-based tapering recommendations, research to improve the understanding of modifiable barriers to GC tapering and increased provider education to raise awareness of the evolving treatment landscape in SLE. Although GCs have been key to SLE management, the introduction of new advanced therapies provides an opportunity to redefine our approach to SLE management and provide patients with improved clinical outcomes without compromising on QOL.

## Data Availability

No new data were generated or analyzed in support of this article.
